# Effects of Mutations on Structure–Function Relationships of Matrix Metalloproteinase-1

**DOI:** 10.3390/ijms17101727

**Published:** 2016-10-14

**Authors:** Warispreet Singh, Gregg B. Fields, Christo Z. Christov, Tatyana G. Karabencheva-Christova

**Affiliations:** 1 Department of Applied Sciences, Faculty of Health and Life Sciences, Northumbria University, Newcastle upon Tyne, NE1 8ST, UK; warispreet.singh@northumbria.ac.uk; 2 Department of Chemistry & Biochemistry, Florida Atlantic University, Jupiter, FL 33458, USA; 3 Department of Chemistry, The Scripps Research Institute/Scripps Florida, Jupiter, FL 33458, USA

**Keywords:** matrix metalloproteinase-1, conformational flexibility, molecular dynamics simulations, mutations, correlated motions

## Abstract

Matrix metalloproteinase-1 (MMP-1) is one of the most widely studied enzymes involved in collagen degradation. Mutations of specific residues in the MMP-1 hemopexin-like (HPX) domain have been shown to modulate activity of the MMP-1 catalytic (CAT) domain. In order to reveal the structural and conformational effects of such mutations, a molecular dynamics (MD) study was performed of in silico mutated residues in the X-ray crystallographic structure of MMP-1 complexed with a collagen-model triple-helical peptide (THP). The results indicate an important role of the mutated residues in MMP-1 interactions with the THP and communication between the CAT and the HPX domains. Each mutation has a distinct impact on the correlated motions in the MMP-1•THP. An increased collagenase activity corresponded to the appearance of a unique anti-correlated motion and decreased correlated motions, while decreased collagenase activity corresponded both to increased and decreased anti-correlated motions.

## 1. Introduction

The matrix metalloproteinases (MMPs) are key enzymes responsible for modulating the balance of collagen and other extracellular matrix (ECM) components in vertebrates. The catabolism of collagen by MMPs is a prerequisite for normal physiological function of cells in vertebrates [1,2,3,4]. MMP-1 has been the subject of a broad range of experimental studies and important conclusions have been drawn about the conformational behavior of MMP-1 domains in solution. The MMP-1 hemopexin-like (HPX) and catalytic (CAT) domains are connected by a linker and exhibit complex conformational motions in order to bind the substrate and perform the chemical reaction of collagenolysis [5,6,7,8,9]. The existence of an equilibrium between the open/extended and closed/collapsed conformation of MMP-1 in solution has been established by experimental methods [8,9,10].

The first molecular dynamics (MD) simulation of MMP-1 bound to a collagen-model triple-helical peptide (THP) [11] provided insight into the role of the linker for modulating conformational dynamics between the HPX and CAT domains and revealed the dynamic nature of interactions between the THP, HPX domain, and CAT domain. The study confirmed the closed or collapsed state of the MMP-1 X-ray crystallographic structure (PDB 4AUO) [7] characterized by close orientation of the MMP-1 HPX and CAT domains. The MD did not cause the MMP-1 conformation to open but allowed the effects of the flexibility on the closed conformation to be explored. Analyses of the dynamics effects including radius of gyration and distances between the centers of masses of the MMP-1 domains were performed [11]. An extensive bioinformatics analysis of 142 MMP X-ray crystallographic structures revealed structural relationships within the enzyme family and structural arrangements important for inhibitor design [12].

**Figure 1:**
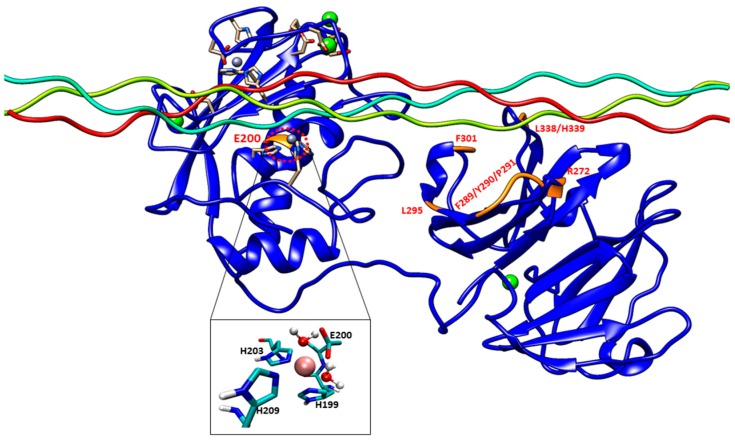
The 3D structure of inactive human matrix metalloproteinase-1 (MMP-1) (E200A mutant) complexed with a triple-helical peptide (THP) (PDB code: 4AUO [7]) drawn using UCSF Chimera [17]. MMP-1 consists of the catalytic (CAT) domain, inter-domain linker, and hemopexin-like (HPX) domain displayed in silhouette round ribbon representation. The THP leading, middle, and trailing strands are shown in tube representation in cyan, green, and red, respectively. The Zn^2+^ and Ca^2+^ ions bound to the enzyme are shown in spherical representation in ice blue and green, respectively. The mutations are shown in orange on the MMP-1•THP structure. The snapshot of the active site of the MMP-1•THP was obtained from molecular dynamics (MD) simulation [11]. The red dotted circle represents the location of the active site in MMP-1. The two solvent molecules are coordinated to the Zn^2+^ along with three His residues with average distance maintained to the X-ray crystallographic distance using harmonic restraints in the simulation.

Experimental studies have identified specific residues from the MMP-1 HPX domain which are involved in interactions with the THP [5,6,7,9]. Kinetic studies identified the effects of the mutations on the enzymatic activity of MMP-1 [5,6,7]. However, there is little understanding of the atomistic effects of the mutations on MMP-1 structure and the impact on MMP-1 flexibility and binding of substrates. The association of the HPX domain with the leading and middle strand of the THP plays a significant role in properly orienting the scissile peptide bond of the leading strand to the CAT domain [9]. Mutagenesis studies on residues from the MMP-1 HPX domain (Figure 1) [5,6,7] indicated effects on enzyme activity, which suggested a long-range conformational effect of the HPX domain and its influence on the ability of the CAT domain to perform effective catalysis. The mutations in MMP-1 range from a single amino acid substitution (E200A) in the CAT domain to a triple amino acid substitution (F289A/Y290A/P291A) in the HPX domain.

The prior MMP-1 mutagenesis studies provided a background to examine the atomistic effects of MMP-1 mutations. MD simulations are applied herein to understand how MMP-1 mutations perturbed the local structure and also to investigate the long-range effects on MMP-1 structure, dynamics, and interactions with a THP. MD simulations have been successfully applied previously to study the effect of mutations on key structural determinants in different enzymes [13,14,15,16]. In order to understand their influence on MMP-1 structure–function relationships, MD simulations were performed on the seven previously described mutants [5,6,7]: E200A, F301Y, F289A/Y290A/P291A, I271A/R272A, L338A/H339A, R272A, and L295S.

## 2. Results and Discussion

### 2.1. Overall Conformational Flexibility of MMP-1•THP with Mutated Residues

**Figure 2:**
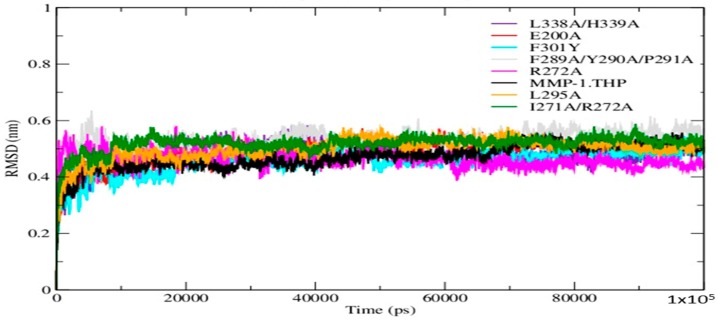
The Root Mean Square Deviation (RMSD) of all Cα atoms of the MMP-1•THP complex in comparison to the mutants.

The Root Mean Square Deviation (RMSD) as a function of time was used to access the structural stability of the simulations. The RMSDs of the mutant forms along with wild-type (WT) MMP-1•THP showed similar trends in that the structures reached equilibration before 20 ns (Figure 2). The average value of RMSD ranged from 5.3 Å (F289A/Y290A/P291A triple mutant) to 4.5 Å (F301Y) (Table S1). The flexibilities of individual residues in the MMP-1•THP complex were assessed by using Root Mean Square Fluctuation (RMSF) analysis (Figure S1; Tables S1 and S2). The basal level fluctuation for WT MMP-1•THP was considered to be 1.4 Å (based upon the mean value), confirmed by a distribution analysis of the RMSFs (Figure S2). 43% of residues showed fluctuations greater than 1.4 Å and 57% of the residues showed fluctuations less than 1.4 Å (Table S1). The RMSF plot of the I271A/R272A and F301Y showed a greater number of residues with fluctuations larger than 1.4 Å in contrast to WT, 60 and 54%, respectively (Table S1). The triple mutant F289A/Y290A/P291A showed the lowest number of residues (29%) with fluctuations >1.4 Å, followed by L338A/H339A (31%) (Table S1). The different mutations induced small changes in the number of hydrogen bonds in comparison to the WT MMP-1•THP (Table S3; Figure S3).

**Figure 3:**
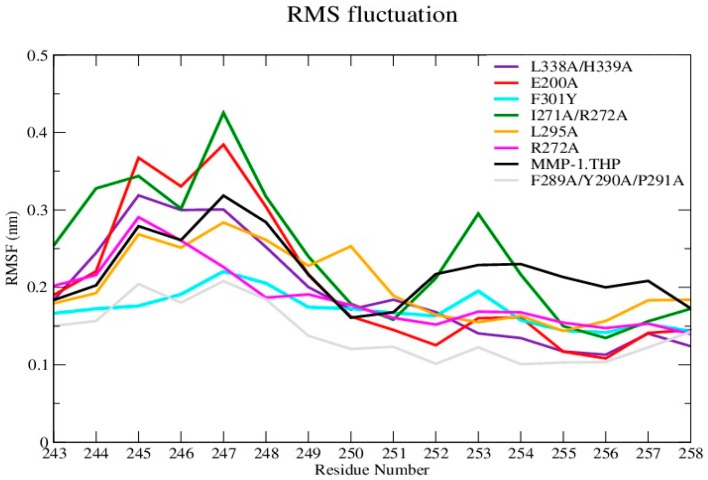
Root Mean Square Fluctuation (RMSF) analysis of linker residues of wild-type (WT) MMP-1•THP and mutants.

The average RMSF value of the linker region in all of the mutants except I271A/R272A showed a slight decrease with respect to WT (Figure 3 and Figure S4A; Table S4). The change in linker flexibility (decrease or increase) could influence the communication between the HPX and CAT domains and the conformational dynamics of the CAT domain and hence contribute to altered enzyme activity. The largest reduction in linker flexibility was seen in the F289A/Y290A/P291A mutant, with an average RMSF value of 1.4 Å in contrast to 2.2 Å for WT MMP-1•THP (Figure 3; Table S4).

Experimental studies have identified specific residues within MMP-1 HPX domain blades I and II (Figure S4B) that interact with the leading and middle strands of the THP [6,7,9]. The study performed by Zhao et al. on MT1-MMP also identified blade I and II residues that interact with the THP [18]. Our recent MD study of MMP-1•THP stressed the importance of HPX domain blade I and blade II residues for interactions with the THP [11]. The majority of the experimental HPX domain mutations are located in blade I, apart from the L338A/H339A mutation which is in blade II [6,7]. The blade I residues constitute the S_10_’ subsite of MMP-1, and mutations in blade I reduced enzymatic activity [5,6,7]. The interactions of HPX domain blade I and II residues with the THP also play a significant role in guiding the CAT domain for effective catalysis of the leading strand of the THP [6,8,9].

RMSF analysis of blade I residue mutants showed relatively higher flexibility of this region for I271A/R272A (Figure S4C and Table S4). The increase in the flexibility of blade I in this mutant with respect to the WT MMP-1•THP could potentially influence THP binding and hence the enzyme activity. Indeed, the I271A/R272A mutation reduced MMP-1 collagenolytic activity [5]. The residues constituting the mutation in blade II showed slightly lower RMSF in comparison to WT MMP-1•THP (Figure S4D). The mutations of residues in blade II (L338A/H339A) resulted in an increase in the enzyme activity [7]. Alteration (decrease or increase) in the flexibility of the linker region would influence the communication between the HPX and CAT domains, binding of the THP, and thus potentially enzyme activity. The specific atomistic mechanism of this effect would need, however, further studies. In addition, strong correlation between the flexibility of residues in blades I and II and the catalytic activity was not extracted from the current data.

**Figure 4:**
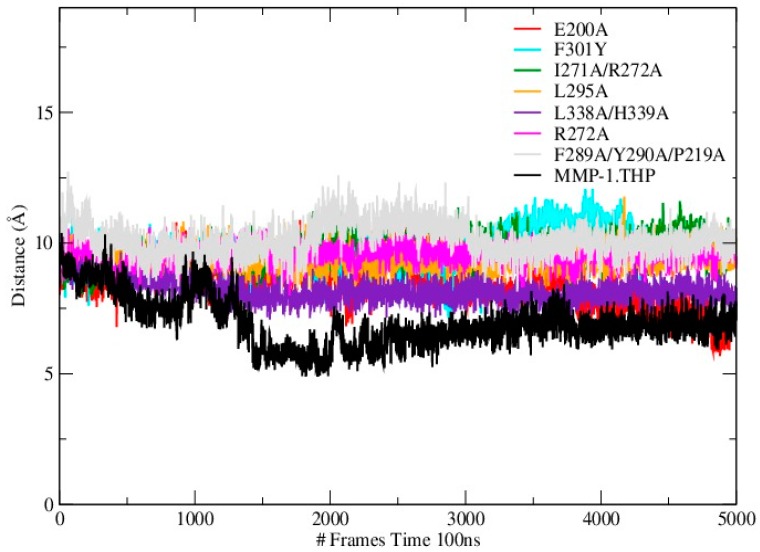
Time evolution of the distance between the scissile bond G775-L776 and Zn^2+^ in WT MMP-1•THP and the mutants.

Mutations can influence not only the local structure, but also regions which are distant from the mutation sites [19]. In order to explore these effects we analyzed the distance between the THP scissile bond and the catalytic Zn^2+^ in all mutants and the WT (Table S3; Figure 4). Importantly, in all mutant simulations we found that the above distance is larger than in the WT, which indicates further that the mutations (all of which but one are in the HPX domain) effect interactions between the scissile bond from the leading chain of the THP and the Zn^2+^ from the MMP-1 CAT domain.

**Figure 5:**
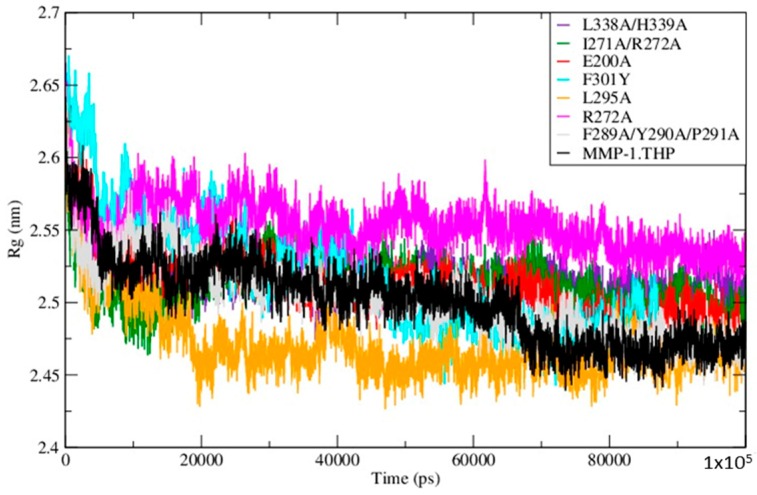
The radius of gyration of the mutants and WT MMP-1•THP.

**Figure 6:**
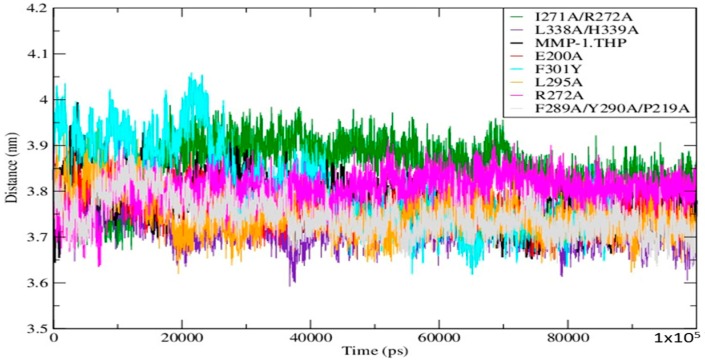
The distance between the center of mass of the CAT and HPX domains comparing the mutant forms and WT MMP-1•THP.

The Radius of Gyration (Rg) of the mutants and WT MMP-1•THP showed similar profiles of structural compression as a function of simulation time (Figure 5; Table S5), with L295A showing the smallest Rg and R272A the largest. The compressions of the structures during simulations of the mutants are consistent with the MMP-1•THP simulation studies [11] and was consistent with the closed or collapsed form of MMP-1 observed in the 4AUO X-ray crystallographic structure [11]. The small differences in the averaged values of the Rg and in the distances between centers of mass between both domains (Figure 6; Table S5) indicate subtle but distinct effects of the mutations on the MMP-1•THP structure and flexibility.

### 2.2. Conformational and Dynamical Effects of Individual Mutations

**Figure 7:**
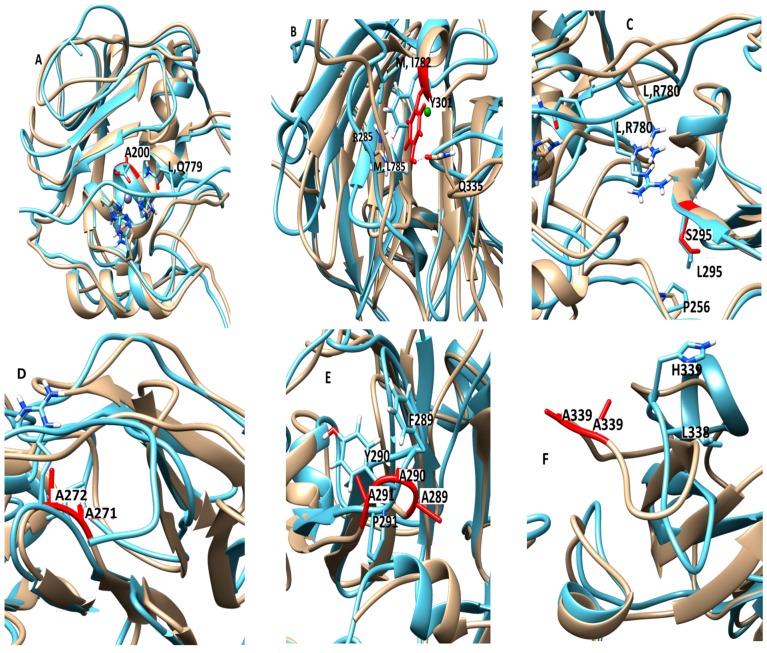
Locations of the mutants in the structure of MMP-1•THP. (**A**) E200A (1.21 Å); (**B**) F301Y (1.24 Å); (**C**) L295S (1.30 Å); (**D**) I271A/R272A (1.3 Å); (**E**) F289A/Y290A/P291A (1.23 Å); and (**F**) L338A/H339A (1.9 Å). The most populated cluster of MMP-1•THP (cyan) is superimposed on the most populated cluster structure from the different mutants (brown) (The RMSD between the mutant structure and the WT MMP-1•THP are indicated in parentheses). Zn^2+^ and Ca^2+^ are shown in spherical representation in ice blue and green, respectively.

The E200A mutation is utilized to greatly suppress the enzymatic activity of MMP-1. The X-ray crystallographic structure of MMP-1•THP (4AUO) incorporated the E200A mutation. In the WT simulations [11], E200 is in close vicinity to the catalytic Zn^2+^ with an average distance of 2.5 Å (Figure 1 and Figure 7A). The carboxylate group of the side chain of E200 interacts with Q779 from the THP leading strand and also makes a hydrogen bond with the N–H group from the backbone of A165 with an average distance of ~3.0 Å (Figure S5A).

In the E200A mutant there were three water molecules which coordinated the active site Zn^2+^ with an average distance of 2.1 Å (Figures S5B and S6A). Two solvent molecules coordinated with the catalytic Zn^2+^ simultaneously made hydrogen bonds with the E200 residue. The interactions between Zn^2+^ and the water molecules were also confirmed by radial distribution analysis (Figures S5C and S6B). The involvement of the water is in agreement with Quantum Mechanics and Molecular Mechanics (QM/MM) studies of the reaction mechanism of MMP-2 [20,21]. The side chain of Q779 (THP leading strand) accessed the active site in a similar manner as in the WT MMP-1•THP [11]. The side chain of A200 in E200A moved away from the catalytic Zn^2+^ with an average distance of ~7.3 Å with respect to 2.2 Å in MMP-1•THP. The backbone of A200 no longer made hydrogen bonds with the backbone of A165 (Figure S6C). The RMSF profile of the E200A mutant did not show any significant difference with respect to MMP-1•THP (Figure S1). However, the loop region between β4–β5 of the CAT domain along with the N-terminal region showed increased fluctuations (Figure S7A). The residues of the linker region (250–259) and β18–β19 (380–390) showed reduced fluctuations compared with MMP-1•THP (Figure S7B–C; Table S2).

**Figure 8:**
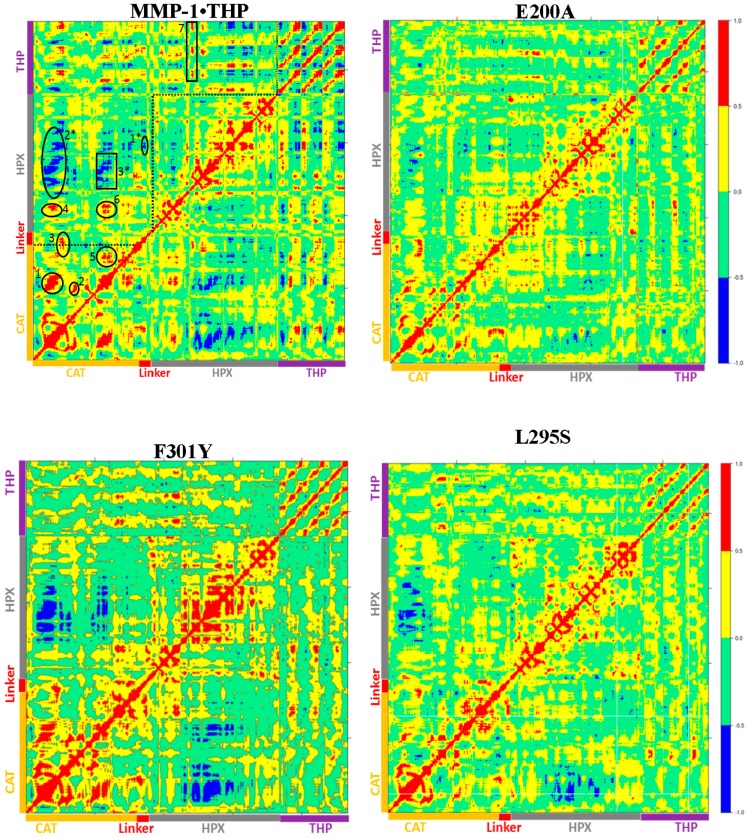

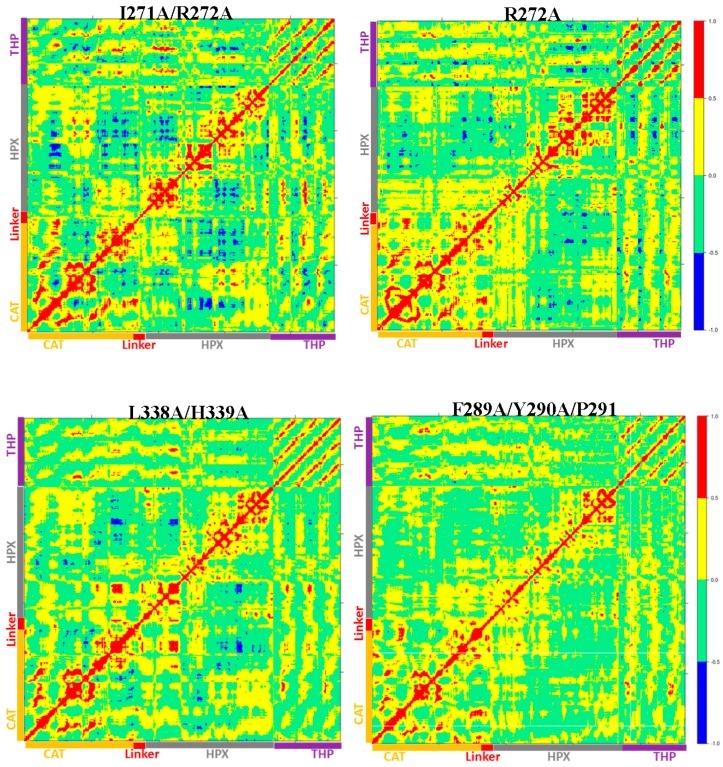
The dynamic cross correlation analysis (DCCA) of MMP-1•THP and mutants. The scale of correlated motion ranges from +1 to −1 and represents positive (red) and negative (blue) motions of Cα atoms. The analysis was performed using Bio3D package [23] in R for WT MMP-1•THP, the areas labeled 1–7 indicate positive correlated motions, while the areas labeled 1*–3* indicate negative correlated motions.

The HPX domain of the E200A mutant showed lower RMSDs with respect to the HPX domain of WT MMP-1•THP (Figure S8). Understanding of the correlated character of atomistic motions in proteins is vital since it relates their structure to function. This insight might be difficult to obtain experimentally, but can be straightforwardly extracted from the MD trajectories [22]. The dynamics cross correlation analysis of the E200A mutant (Figure 8) showed correlated motion between the strands of the THP as was observed for MMP-1•THP. However, there was no negative correlated motion seen in the THP strand as was observed for WT MMP-1•THP. An important difference seen in the E200A mutant was that the THP strands showed very low positive correlated motion towards the CAT domain. There was positive correlated motion seen between the THP and the HPX domain. The extents of both positive and negative correlated motion were reduced in the E200A mutation. Thus, although commonly used to provide a locally reduced enzyme activity, the E200A mutation has long range effects.

The F301Y mutation site is located between the loop region of D α-helix and β10 of the HPX domain blade I (Figure 1 and Figure 7B, and Figure S9), part of an MMP-1 subsite for collagen binding. In the MD simulation of the WT MMP-1•THP, F301 exhibited hydrophobic interactions with the side chains of I782 and L785 from the THP middle strand, with average distances of 4.3 and 4.1 Å, respectively (Figure S10). This result is inconsistent with the X-ray crystallographic structure but supports results from NMR spectroscopic experiments [6,7,8,9]. The hydroxyl group of Y301 in the F301Y mutant forms hydrogen bonds with the side chains of R285 (2.5 Å) and Q335 (3.1 Å) of the HPX domain. The backbone of L785 also forms a hydrogen bond with the side chain of Y301, with an average distance of 3.5 Å. I782 and L785 from the THP show weaker hydrophobic interactions in the F301Y mutant compared with WT MMP-1•THP. The RMSF analysis of F301Y showed increased fluctuations of residues from blade III between β15-D-β16 (Figure S11A). The linker region of the F301Y mutant showed reduced fluctuations in comparison to the linker region of WT MMP-1•THP (Figure S11B). Residues 294–310 from blade I that belong to the loop region between β9-d-β10, in the vicinity of the F301Y mutation also showed increased flexibility (Figure S11C). This region is in very close contact with the THP leading strand and makes important binding interactions with the THP as experimentally demonstrated by Arnold and co-workers [6]. The RMSD of the CAT and HPX domains of the F301Y mutant showed lower structural deviation in comparison to MMP-1•THP (Figure S12). The F301Y mutant in the study performed by Arnold and co-workers [6] had a Radius of gyration (Rg) value (33.6 Å) which was 18% higher compared with WT MMP-1 (Rg = 28.5 Å). These results suggest disruption of the CAT/HPX domain interface in the F301Y mutant due to destabilization of collagen interactions mediated by blade I residues of the HPX domain [6]. The differences between the Rg of the F301A mutant in comparison to WT MMP-1•THP in our study are smaller (due to the length of the simulation) but still indicated a similar trend.

The dynamic cross correlation analysis (DCCA) of F301Y showed relatively strong anti-correlation between the CAT and HPX domain residues in comparison to MMP-1•THP and slightly reduced positive correlation overall (Figure 8). This is an indication of the sensitive effect of the F301Y mutation on the interactions with the THP and motions of the HPX domain. The F301Y mutant exhibited a ~90% reduction in collagenase activity [7]. The F301Y mutation also showed a 30-fold decrease in THP binding by MMP-1 [6], which was proposed to be due to the absence of interaction of the F301 side chain with the THP molecule rather than large conformational changes in the HPX domain.

The L295S mutation is located in the β9 region of the HPX domain (Figure 7C). In the MD of WT MMP-1•THP the backbone of L295 forms a hydrogen bond with the side chain of R780 of the THP leading strand with an average distance of 3.5 Å. The residues in the vicinity of L295, such as N296 and E294, also have interactions with the THP leading strand [11]. The side chain of S295 in L295S formed new interactions with the backbone of P256 of the linker region with an average distance of 4.1 Å (Figure S13). The hydrogen bond with R780 from the THP leading strand in WTMMP-1•THP is not present in L295S (Figure S13).

The CAT domain of L295S showed slightly increased fluctuations with respect to WT MMP-1•THP (Figure S14A,B), while the HPX domain exhibited reduced fluctuations in the region encompassing β17–β18 (Figure S14C). The linker region residues joining the HPX domain also showed slightly reduced flexibility in comparison to WT MMP-1•THP (Figure S14D). The DCCA analysis of L295S revealed some reduction in both positive and negative motion with respect to MMP-1•THP. The triple-helix strands lost the correlated motion with the HPX domain and showed correlated motion towards the CAT domain (Figure 8) which indicated potential influence on THP binding by the HPX domain in this mutant. The L295S mutant showed approximately 60% reduction in collagenase activity in comparison to MMP-1 [7].

The R272A and I271A/R272A mutations are located in the blade I region (S_10_’ subsite) of the HPX domain (Figure 7D). R272 has extensive interactions with the THP middle strand in MMP-1•THP [7,11]. The side chain and the backbone of R272 make hydrogen bonds with the backbone and side chain of O786 and R789, respectively. The aliphatic region of the R272 side chain is involved in formation of hydrophobic interactions with the side chains of R789 and L785 of the THP middle stand [7,11]. The backbone of I271 forms hydrogen bonds with the backbone of E274 and T269, and the side chain of I271 participates in hydrophobic interactions with the side chain of V321 of blade II [7,11]. In the R272A mutant, the side chain of A272 made hydrophobic interactions with the side chains of L785 and Q788 of the THP middle strand with average distances of 4.1 and 4.2 Å, respectively (Figure S15A); however, there are no hydrogen bonds with O786 and R789 (Figure S15B). The RMSF of the residues in the vicinity of R272A (271–276; the loop between β6–β7 of blade I) showed an increased fluctuation in comparison to WT MMP-1•THP (Figure S16A). These residues interact with the THP [6]. The residues harbouring the active site in the CAT domain (210–215; loop between the B–C helix) showed slightly increased fluctuations in contrast to WT MMP-1•THP (Figure S16B). R272A showed a slight increase in its average Rg (25.5 Å) in comparison to WT MMP-1•THP (24.8 Å).

The I271A/R272A mutant (Figure 7D) showed an increase in the RMSF of blade I residues ranging from 275-285 (β7–β8) and 295-310 (loop region β9–β10) (Figure S17) and also increased flexibility seen in residues 345–355 of blade II of the HPX domain (Table S2). The extent of anti-correlated motions in I271/R272 is similar to WT MMP-1•THP and new anti-correlated motion emerged (Figure 8). The HPX domain showed anti-correlated motions between the CAT domain and strands of the THP. The linker region exhibited anti-correlated motion towards the HPX domain. New correlated motions between the HPX domain and the CAT domain appeared. The R272A mutant showed a similar profile to dynamic cross correlated motions of I271/R272A, but with smaller magnitudes (Figure 8).

R272A and I271A/R272A mutations have the most significant effect on the enzyme activity of MMP-1. The I271A/R272A mutant has less than 10% of the collagenase activity of WT MMP-1 [5,7]. Analysis of individual kinetic parameters for R272A and I271A/R272A hydrolysis of a fluorogenic THP (fTHP-17) revealed that *K*_M_ increased and *k*_cat_ decreased compared with WT MMP-1 [5]. Since both *K*_M_ and *k*_cat_ changed, the R272A and I271A/R272A mutations affected more than just substrate binding. It was suggested that both substrate binding and coupled motions for catalysis were altered by these mutations [5]. The present MD study has identified the long range effects of R272A and I271A/R272A mutations within MMP-1•THP, which was primarily new anti-correlated motions (Figure 8).

The F289A/Y290A/P291A triple mutant (Figure 7E) was next considered. The interactions in the X-ray crystallographic structure of residues Y290 and F289 were stable in the MD simulation of the WT MMP-1•THP [11]. The triple mutant showed the very lowest RMSF overall as compared to other mutants in relation to WT MMP-1•THP (Table S1). The cross correlation analysis showed very low anti-correlated motion observed in F289A/Y290A/P291A (Figure 8). There was also a significant reduction in the positive correlation motions with respect to WT MMP-1•THP. The THP strands showed positive correlated motion towards the residues of the CAT domain. F289A/Y290A/P291A had ~30% reduction in collagenase activity compared with WT MMP-1 [7].

The last mutant considered was L338A/H339A. L338 and H339 are located on the loop between β13 and β14 of the HPX domain (blade II) and are on average 17 Å away from the THP (Figure 7F). The X-ray crystallographic structure and MD simulations of the WT do not reveal significant interactions of these residues with the THP. The average RMSF of this mutant was slightly lower than the WT (Table S1). The DCCA of L338A/H339A (Figure 8) showed significantly lower anti-correlated motions with respect to WT MMP-1•THP. Residues 100–120 of the CAT domain showed limited negative correlation against the HPX domain β-sheets (residues 310–380). Residues 175–185 of the CAT domain also showed reduced negative correlation towards residues 330–350 of the HPX domain. However, the L338A/H339A mutation also results in an anti-correlation between the C-terminal region of the CAT domain and the N-terminal region of the HPX domain that is not observed in the WT or other mutant enzymes (Figure 8). The anti-correlation may be a shift of an anti-correlation in the WT enzyme (noted as 6 in Figure 8) further towards the C-terminal region of the CAT domain. A unique small correlation was observed between the C-terminal regions of the CAT and HPX domains in the L338A/H339A mutant enzyme (Figure 8). The combined L338A/H339A mutation increased the collagenase activity of MMP-1 by approximately 10% [7]. The change in the anti-correlated motions resulting from the L338A/H339A mutation may play a factor in increased collagenase activity.

The changes in the anti-correlated motions can be complex and not unidirectional. In the case of L338A/H339A, a decrease in previously observed anti-correlations and the appearance of a unique anti-correlation are accompanied by increased enzyme activity, while in F289A/Y290A/P291A a virtual complete lack of anti-correlations (as well as decreased correlated motions) are related to decreased enzyme activity. Further studies, including simulations at longer time scales, would be necessary for more detailed analysis of these effects.

It is important to note that the X-ray crystallographic structure of MMP-1•THP [7] does not represent the productive enzyme•substrate (E•S) complex. In contrast, NMR studies have reported a productive complex [9]. Nevertheless, the X-ray crystallographic structure of MMP-1•THP is still the only available structure that presents the atomistic interactions between the enzyme and substrate (the NMR-derived structure is based on docking the THP). Both the X-ray crystallographic and NMR structures of MMP-1•THP are in agreement about most of the THP interactions with the HPX domain and the closed conformation of MMP-1. The structures differ in the position of the THP in the CAT domain, where the NMR-derived structure represents a productive complex, while the X-ray crystallographic structure does not. Most of the mutations are located in the HPX domain, therefore the MD simulations provide relevant insight on the influence of these mutations on the MMP-1•THP structure and dynamics. In the absence of experimental structures of the mutants (with the exception of E200A), the present study also provided an atomistic view of short- and long-range effects and their impact on the correlated motions of enzyme-substrate complex structures. The effects of the mutations on the different X-ray crystallographic and NMR structures would be the subject of further comparative MD studies.

## 3. Materials and Methods

The MMP-1•THP X-ray crystallographic structure (PDB ID 4AUO) [7] was used for the present simulations and prepared as described previously [11]. The seven mutant structures were prepared with the Modeller program [24]. MD simulations were performed using Gromacs 4.5.5 package [25,26,27] with GROMOS96 43a1 force field [28]. The energy minimization was performed to remove steric clashes in the X-ray crystallographic structure first by using the steepest descent [29] and then by using conjugate gradient algorithm [30]. The MD setup was performed based upon the methodology described previously [11]. The productive MD was carried out using NPT (constant Number of atoms, Pressure and Temperature) ensemble and the initial velocities for the MD simulation were drawn from the Maxwell velocity distribution at 300 K. The productive simulations were carried out for 100 ns. The analyses of the trajectories were performed using tools from the Gromacs software package for the equilibrated parts only (20–100 ns). The visualization of MD trajectories and the structures were performed using Visual Molecular Dynamics (VMD) [31] and University of California at San Francisco UCSF Chimera [17] software. The Bio3D package [23] in R was used to produce domain cross correlation maps.

## 4. Conclusions

Experimental studies show that a variety of mutations in the HPX and CAT domains of MMP-1•THP have strong effects on the enzyme activity. The present computational studies provide atomistic explanation of these effects and reveal that the mutations have not only local structural effects but also long-range impact on the protein structure, dynamics, and the interactions with a triple-helical substrate. The mutations change the flexibility around the local site but also influence distant regions from the HPX domain, the linker region, and the CAT domain. In addition, the mutations modulate the intensity and the nature of the correlated and anti-correlated motions. An increased collagenase activity in L338A/H339A mutant corresponded to the appearance of a unique anti-correlated motion and decreased correlated motions, while decreased collagenase activity in the other mutations corresponded both to increased and decreased anti-correlated motions. Our studies provide important structural and dynamic information which correlates and helps to explain the experimentally measured effects of the mutations. With the exception of the E200A mutant, there are no X-ray crystallographic structures of MMP-1•THP with mutated residues, and the present study provides this missing structural information and asserts that the mutations have delicate, distinct, and specific effects on the structure, interactions, and dynamics in MMP-1•THP. The magnitude of the changes in the local interactions and dynamics are in agreement with the effects of mutations in other proteins [13,14,15,16].

## Supplementary Materials

Tables S1–S5 and Figures S1–S17 are available online at www.mdpi.com/1422-0067/17/10/1727/s1.

## Abbreviations

MD	Molecular Dynamics
MMP-1	Matrix Metalloproteinases-1
THP	Triple Helical Peptide
RMSD	Root Mean Square Deviation
