# Supplementary material for: Effects of Mutations on Structure–Function Relationships of Matrix Metalloproteinase-1
Source: Int J Mol Sci. 2016 Oct 14;17(10):1727. doi: 10.3390/ijms17101727 (PMC5085758; doi:10.3390/ijms17101727)
Supplement: Supplementary file 1 [file ijms-17-01727-s001.pdf]

Supplementary Materials: Effects of Mutations on Structure–Function Relationships of Matrix Metalloproteinase-1

Warispreet Singh, Gregg B. Fields, Christo Z. Christov and Tatyana G. Karabencheva-Christova

Table S1. Average Root Mean Square Deviation (RMSD) and Root Mean Square Fluctuation (RMSF) of wild type and mutant MMP-1•THP.

MMP-1	Average RMSD	RMSD Standard Deviation	RMSF > 1.4 (Å) (%)	RMSF < 1.4 (Å) (%)	Location of Mutation
WT	4.7	0.44	43	57	–
F289A/Y290A/P291A	5.3	0.35	29	71	β8–β9 (HPX)
R272A	4.6	0.29	43	57	β6–β7 (HPX)
L338A/H339A	4.7	0.41	31	69	β13–β14 (HPX)
F301Y	4.5	0.42	54	46	D-β10 (HPX)
L295S	4.9	0.39	47	53	β9 (HPX)
I271A/R272A	5.2	0.92	60	40	β6–β7 (HPX)
E200A	4.8	0.44	38	62	B helix (CAT)

Table S2. RMSFs of the structural elements in MMP-1 mutants compared with WT MMP-1•THP. An increase or decrease is based on an average change in RMSF of > 0.3 Å in at least 50% of the residues.

Mutant	Increased		Decreased	
	Residue Span	Structural Location	Residue Span	Structural Location
F289A/Y290A/P291A	–	–	100–128, 146–158, 225–237, 245–258, 319–338, 382–391	β1-A, β3–β4, Linker, E-β11–β12–β13, β17–β19
R272A	81–92, 210–215, 271–280, 350–355, 430–435	N-terminus, B-C, β6–β7, F-β15, G-β23	250–258,148–158, 180–189	Linker, β3–β4, β5-B
I271/R272A	81–90, 275–285, 295–310, 345–355	N-terminus, β7–β8, β9-D, F-β15	–	–
L338A/H339A	168–172, 288–292	β4–β5, β8–β9	100–109, 147–157, 252–263, 377–391	β1-A, β4–β5, Linker, β17–β19
F301Y	81–90, 168–173, 187–192, 296–309, 364–368	N-terminus, β4–β5, β5-B, β9-D-β10, β15–β16	250–262	Linker
L295S	81–92, 167–172, 206–223, 252–258	N-terminus, β4–β5, B-C, Linker	375–390	β17–β18
E200A	168–173, 83–87	β4–β5, N-terminus	250–259, 380–390	Linker, β18–β19

Orange = CAT domain, Green = HPX domain, Red = Linker region.

Table S3. Average number of hydrogen bonds and average distance between the scissile bond and catalytic Zn^{2+}.

Enzyme	Average Number of H-Bonds	Average Distance between the Scissile Bond and Catalytic Zn^{2+} (Å)
WT	296	6.9
F289A/Y290A/P291A	295	10.1
R272A	293	9.5
L338A/H339A	301	8.1
F301Y	300	9.7
L295S	299	9.3
I271A/R272A	300	9.6
E200A	301	8.4

Table S4. The average RMSF value of the linker, blade I and blade II regions, and HPX and CAT domains of mutants in comparison to MMP-1•THP. μ = mean RMSF and σ = standard deviation.

MMP-1	μ Linker	σ Linker	μ Blade I	σ Blade I	μ Blade II	σ Blade II	μ HPX Domain	μ HPX Domain	μ CAT Domain	σ CAT Domain
WT	2.2	0.4	1.0	0.5	1.3	0.4	1.3	0.5	1.1	0.3
F289A/Y290A/P291A	1.4	0.3	0.9	0.9	1.1	0.4	1.2	0.4	0.9	0.2
R272A	1.8	0.4	1.1	0.5	1.3	0.5	1.4	0.6	1.1	0.2
L338A/H339A	1.9	0.6	1.1	0.6	1.1	0.4	1.2	0.5	1.1	0.3
F301Y	1.7	0.2	1.1	0.3	1.5	0.3	1.4	0.4	1.3	0.3
L295S	2.0	0.4	1.1	0.3	1.2	0.4	1.2	0.4	1.3	0.5
I271A/R272A	2.4	0.8	1.3	0.4	1.6	0.6	1.5	0.5	1.2	0.4
E200A	2.0	0.9	1.0	0.4	1.2	0.4	1.2	0.4	1.1	0.3

Table S5. Average value of radius of gyration and centre of mass for MMP-1•THP and mutants.

MMP-1	Radius of Gyration (Å)	Center of Mass (Å)
WT	24.8	37.7
F289A/Y290A/P291A	25.0	37.5
R272A	25.5	38.0
L338A/H339A	25.2	37.2
F301Y	25.2	38.1
L295S	24.7	37.5
I271A/R272A	25.1	38.5
E200A	25.1	37.6

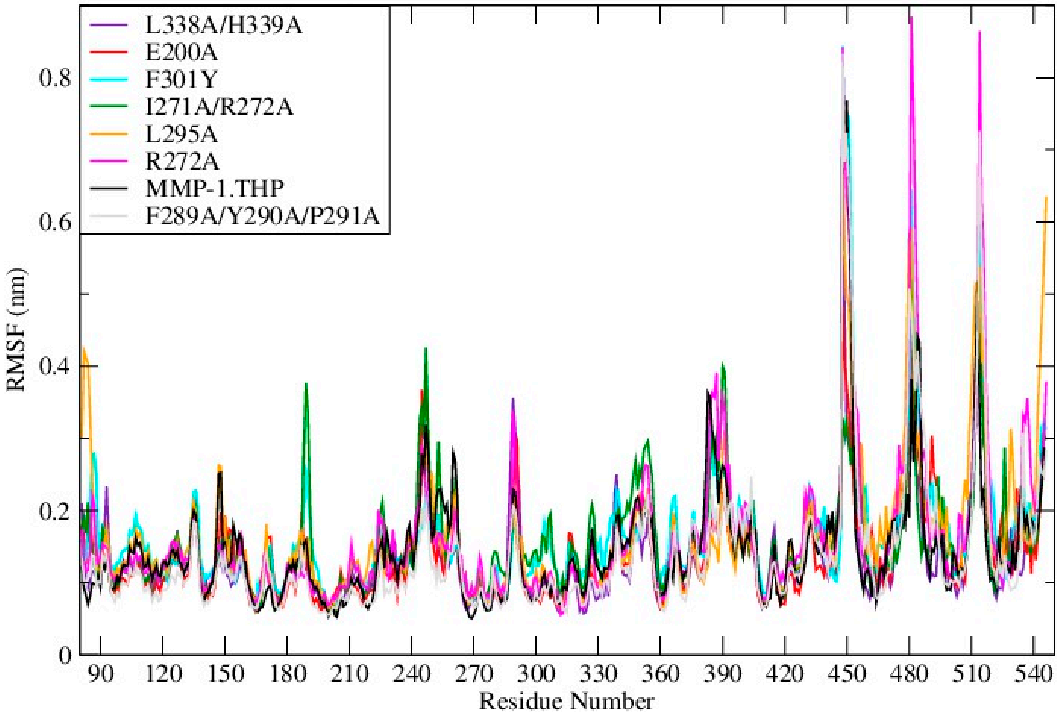

Figure S1. RMSF profiles of the mutants in comparison to the WT MMP-1•THP.

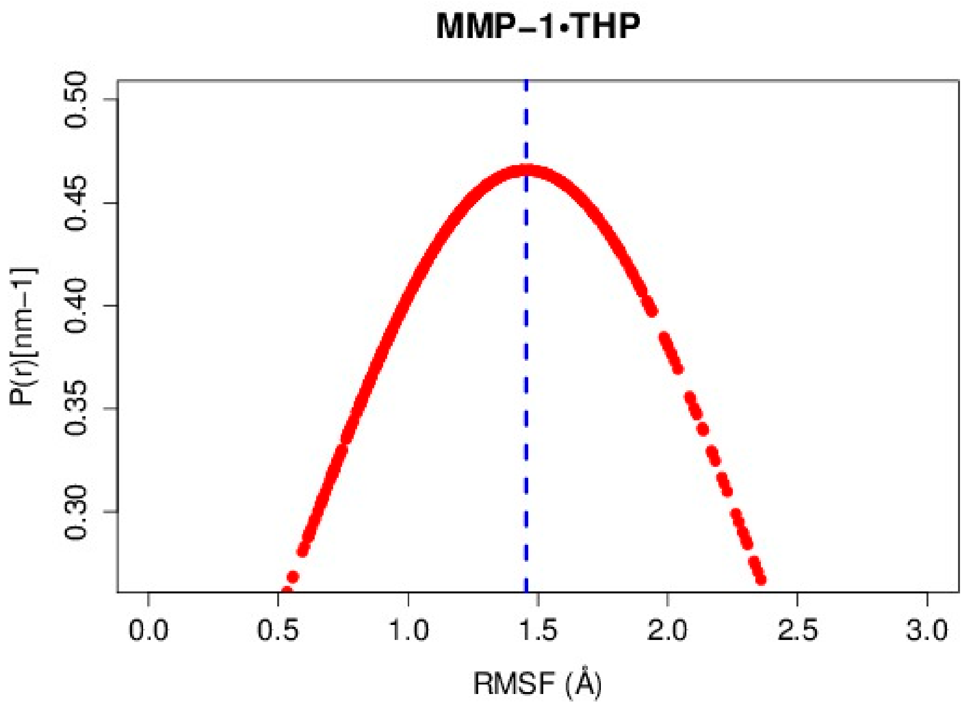

Figure S2. Distribution of the RMSF of MMP-1•THP. The blue line indicates the mean value of the distribution (1.4 Å).

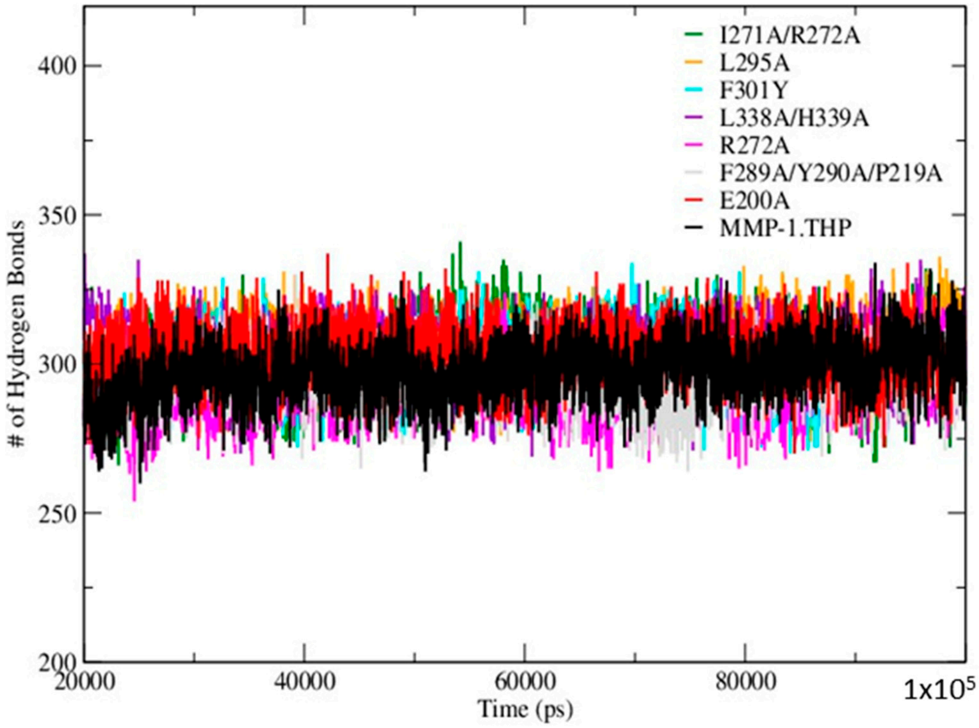

Figure S3. Time evolution of the hydrogen bonds in MMP-1•THP and the mutants.

(**A**)

(**B**)

(**C**)

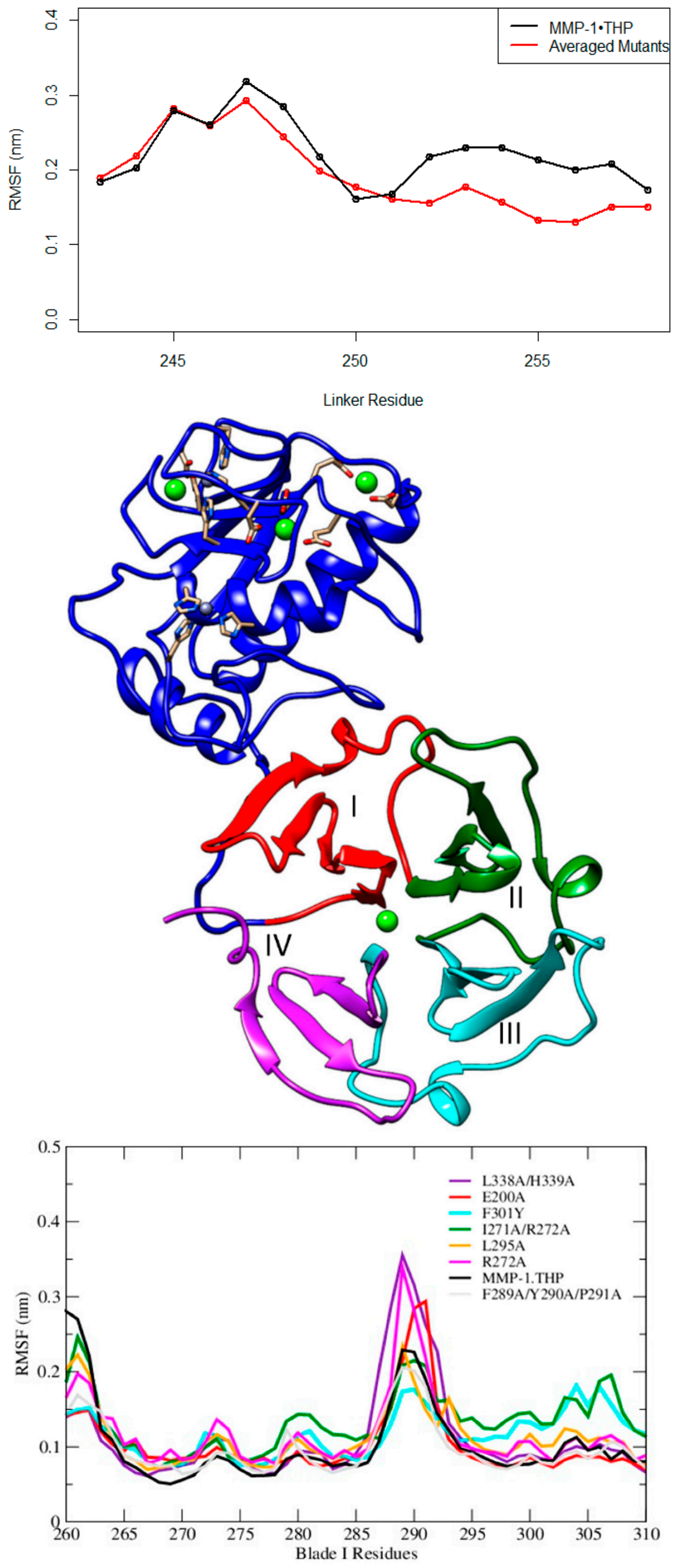

Figure S4. *Cont.*

(**D**)

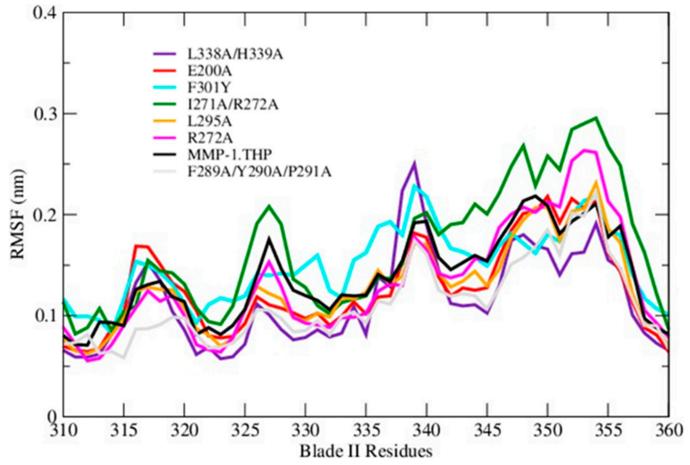

Figure S4. RMSF analysis of the linker and HPX domain blade I and blade II. (**A**) Averaged RMSF analysis of the linker for WT MMP-1•THP and all mutants; (**B**) The numbering of blades of the MMP-1 HPX domain; (**C**) RMSF analysis of blade I in WT MMP-1•THP and all mutants; (**D**) RMSF analysis of blade II in WT MMP-1•THP and all mutants.

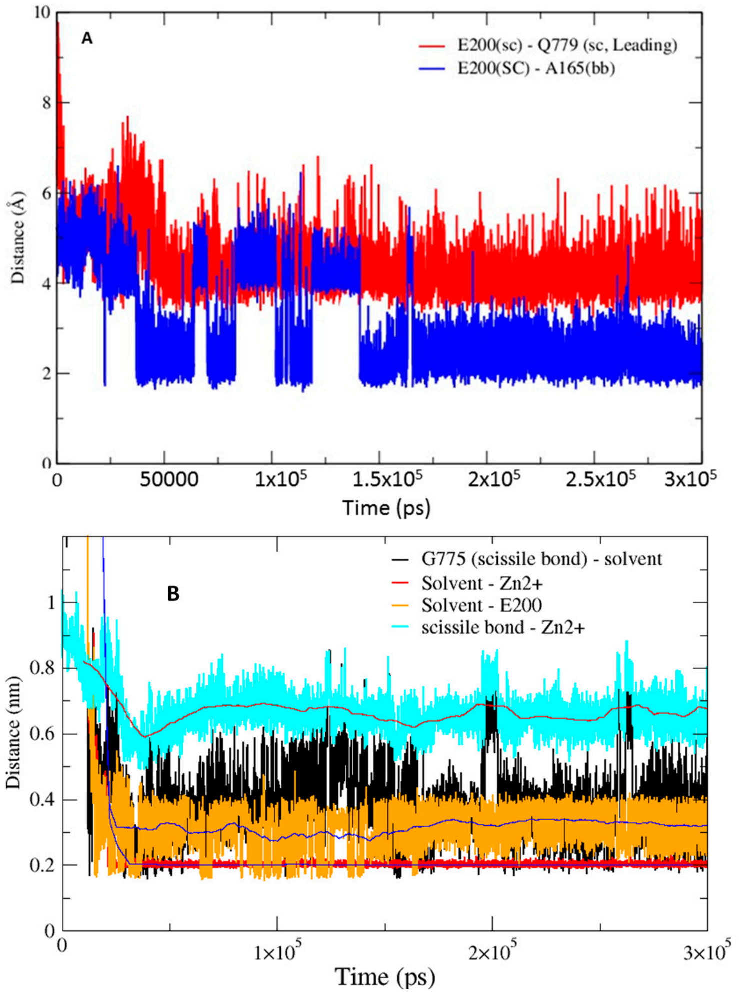

Figure S5. *Cont.*

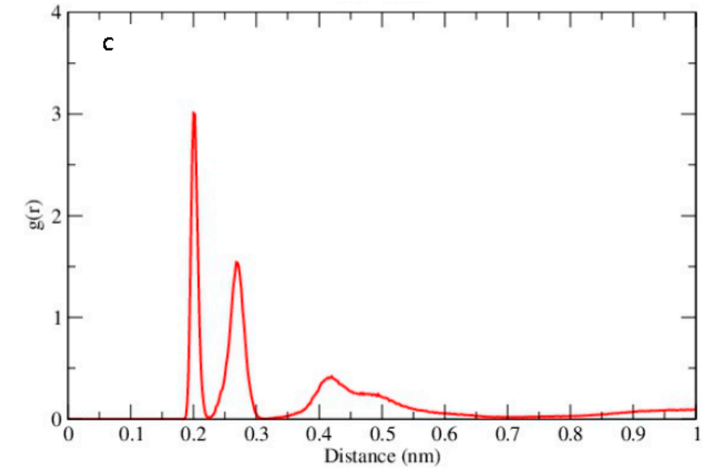

Figure S5. Interactions of E200 of MMP-1•THP. (**A**) The E200 residue interactions with the THP leading strand and forms hydrogen bonds with the backbone of A165; (**B**) The solvent molecule interactions with E200 and the catalytic Zn^{2+} in the active site. The distance between the center of mass of the side chain and the backbone were used in this analysis; (**C**) The radial distribution functions g(r) of the catalytic Zn^{2+} showing the distribution of solvent molecules for 300 ns trajectory in MMP-1•THP.

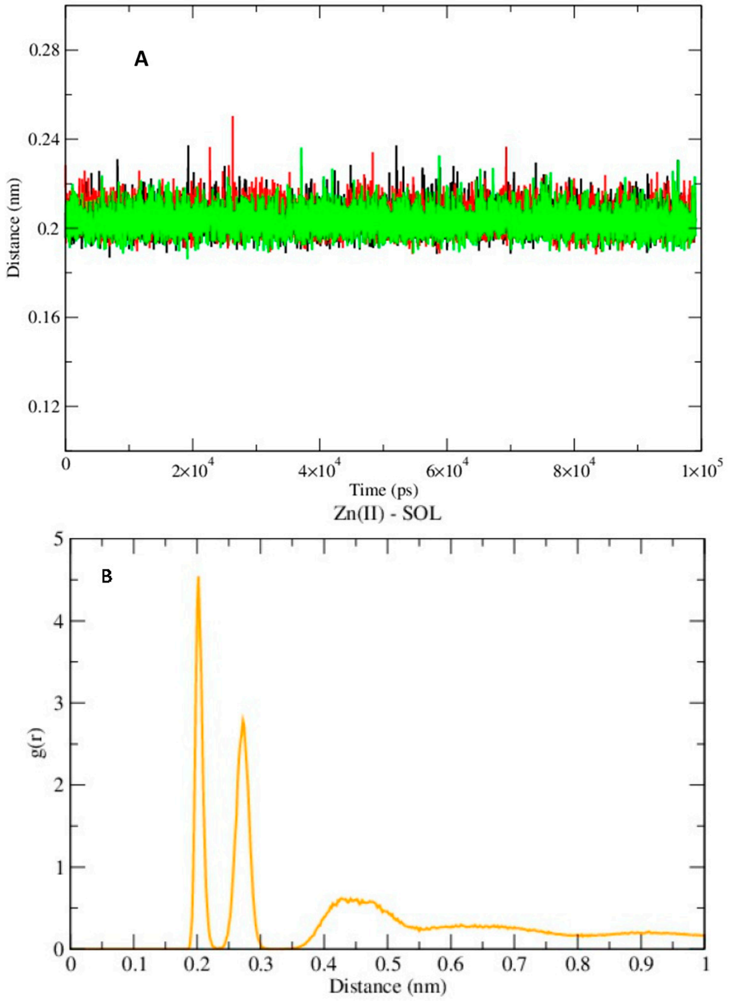

Figure S6. *Cont.*

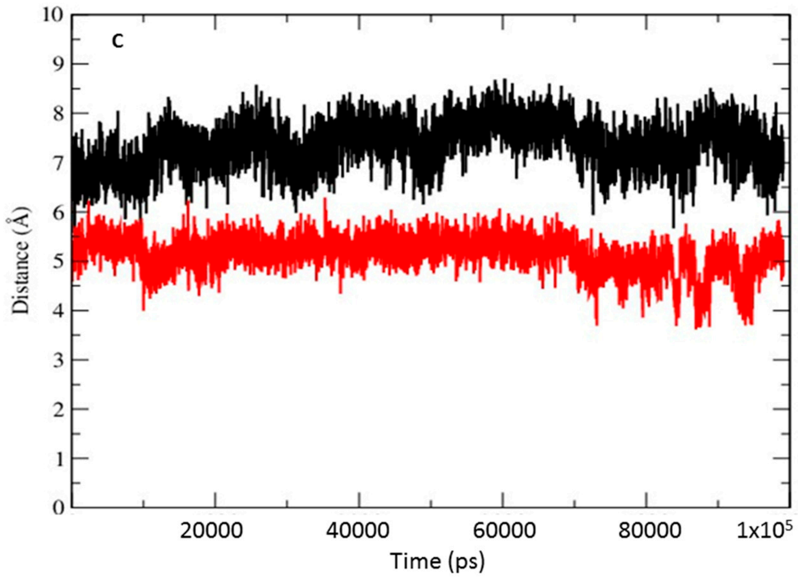

Figure S6. Interactions of the E200A mutant. (**A**) The interactions of the solvent molecules with the catalytic Zn^{2+} in E200A (solvent 1-Zn^{2+} in black, solvent 2-Zn^{2+} in red and solvent 3-Zn^{2+} in green); (**B**) The radial distribution functions g(r) of the catalytic Zn^{2+} showing the distribution of solvent molecules for 100 ns trajectory; (**C**) The distance between the side chain and backbone of A200 with respect to the catalytic Zn^{2+} (black) and A165 backbone (red).

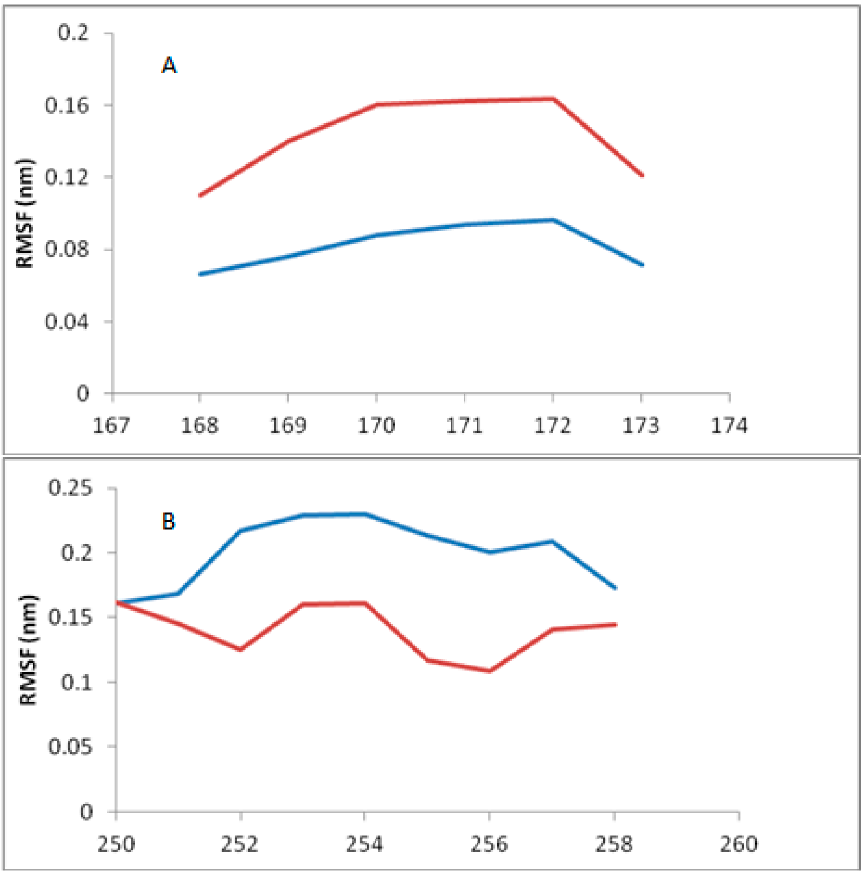

Figure S7. *Cont.*

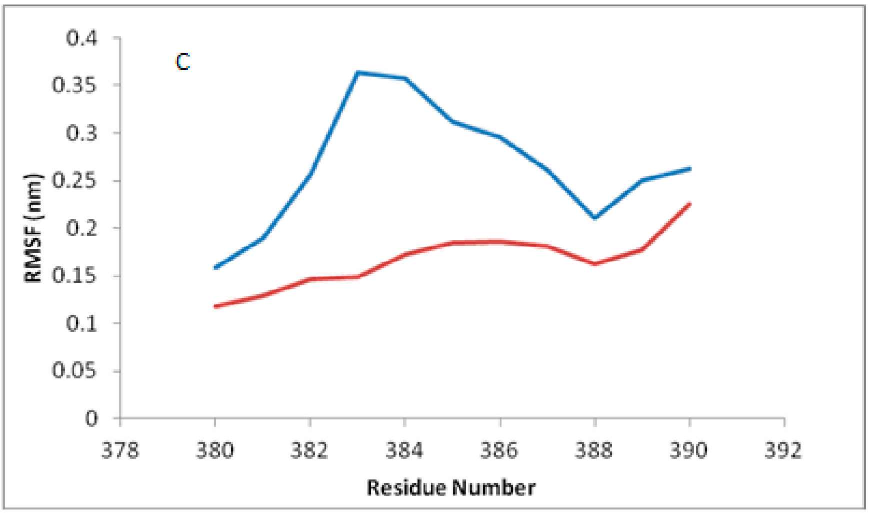

Figure S7. The RMSF of regions of E200A (red) in comparison to WT MMP-1•THP (blue). (**A**) Residues 167–174 belonging to the loop region of β4–β5 of the CAT domain. Residues on this loop coordinate to the structural Zn^{2+}, particularly D175; (**B**) Residues 250–260 belong to the linker region; (**C**) Residues spanning 378–392, part of blade III of the HPX domain and belonging to β18–β19.

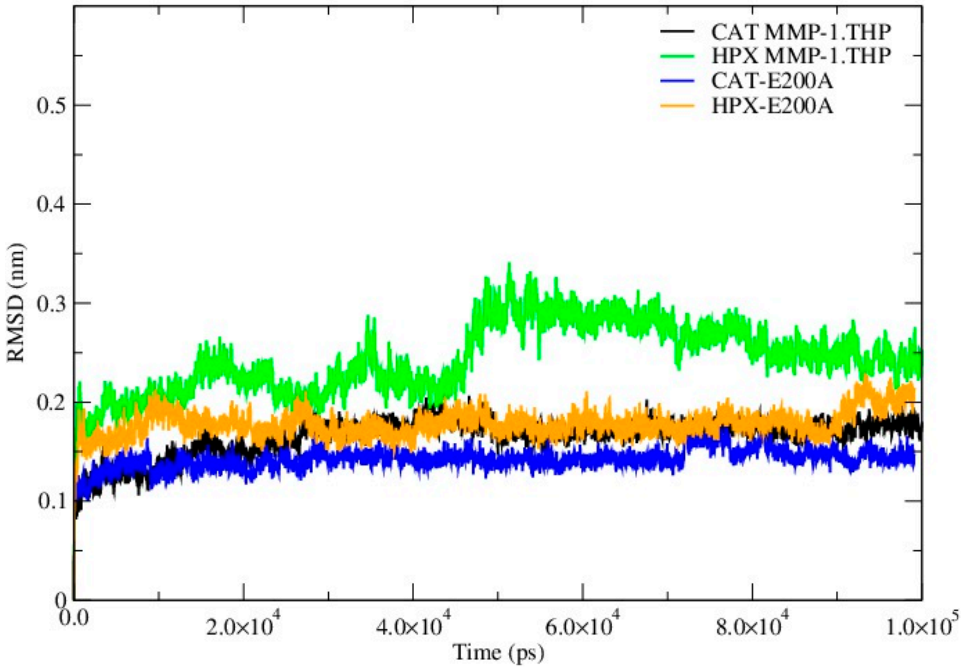

Figure S8. The RMSD of the CAT domain, HPX domain, and linker region of E200A in comparison to the WT MMP-1•THP domains using Cα atoms.

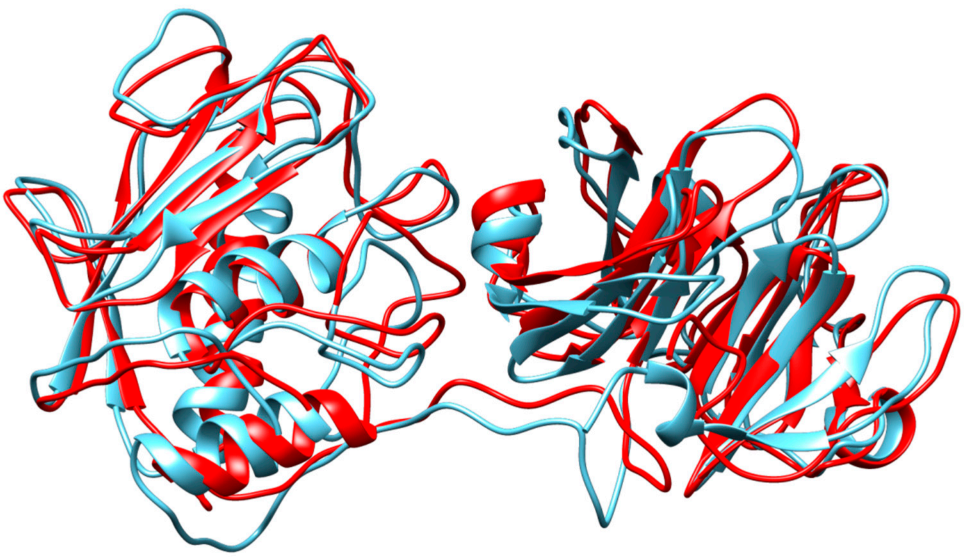

Figure S9. The superposition of most populated cluster of F301Y mutant (red) and WT MMP-1•THP (cyan). There is a clear change in the conformation of blade I in the F301Y mutant compared with WT MMP-1•THP.

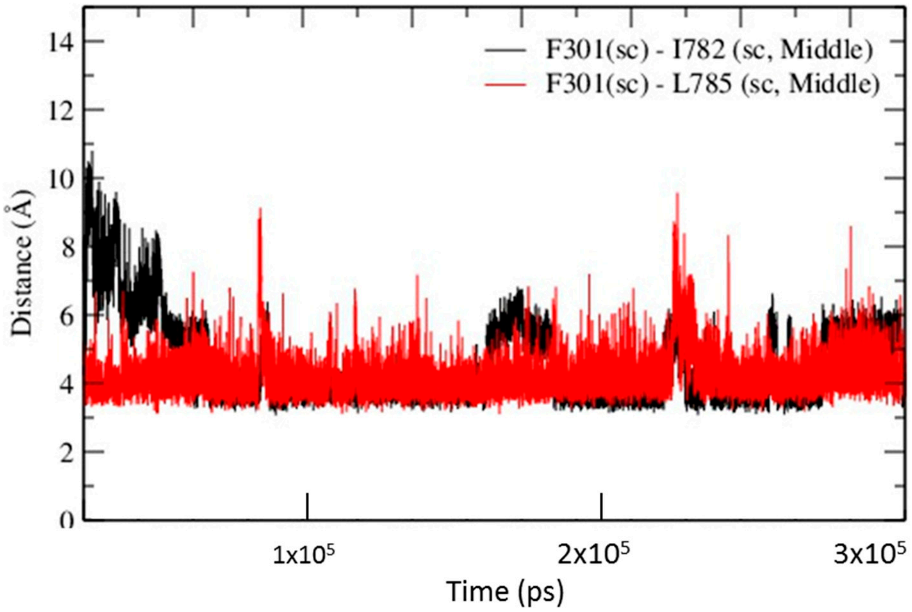

Figure S10. Interactions of HPX domain F301 with the THP middle strand in WT MMP-1•THP.

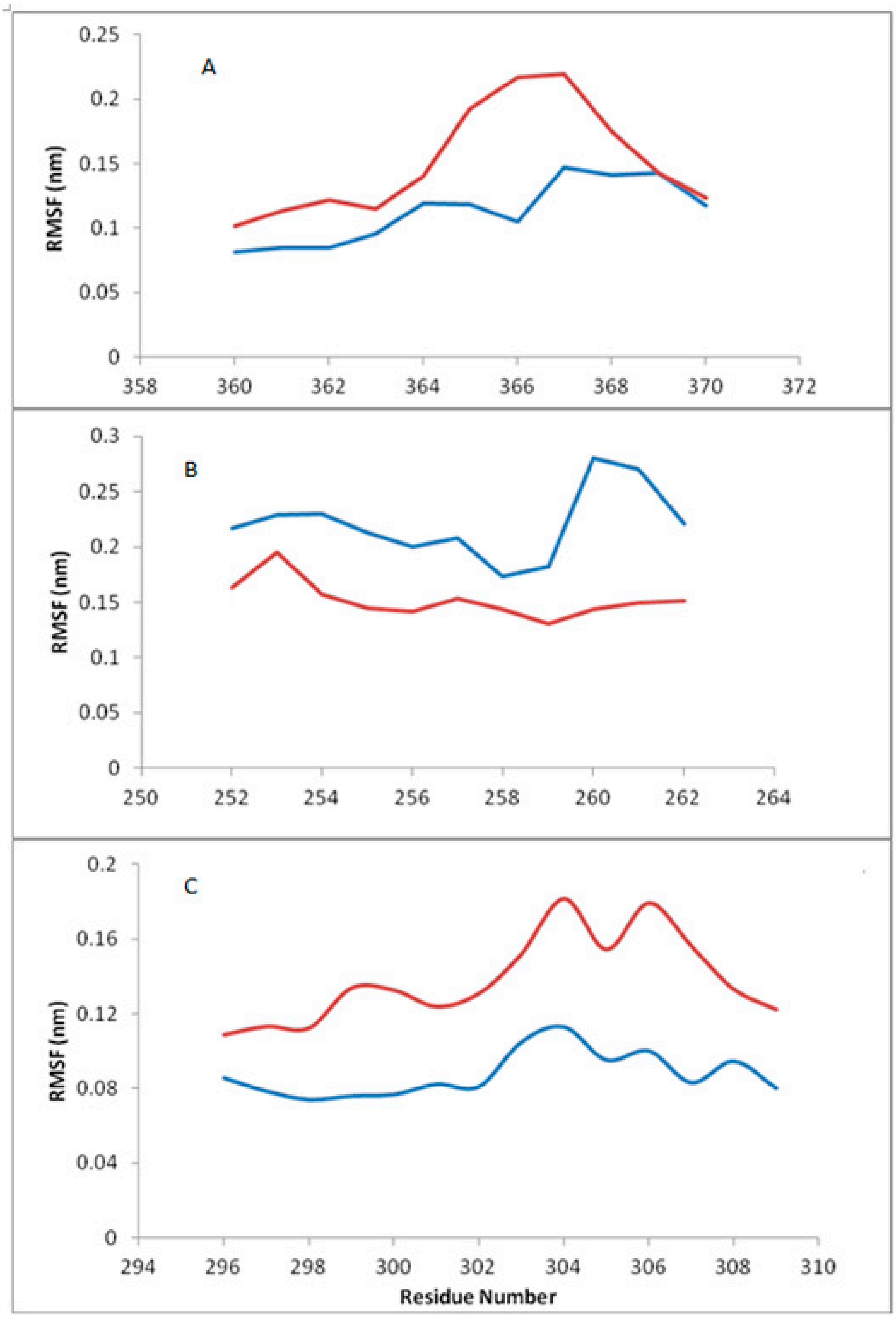

Figure S11. The RMSF analysis of the F301Y mutant (red) and WT MMP-1•THP (blue). (**A**) The residues spanning 360–372 belong to blade III of the HPX domain (β15–β16); (**B**) Residues 250–264 belong to the linker region; (**C**) Residues 294–310 belong from blade I of the HPX domain.

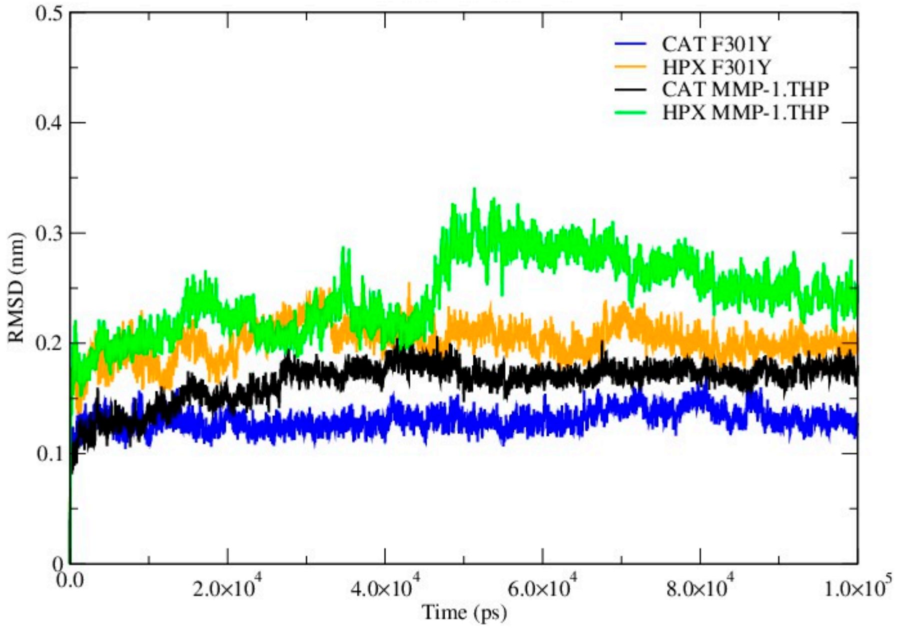

Figure S12. The RMSD of individual domains of the F301Y mutant in comparison to WT MMP-1•THP.

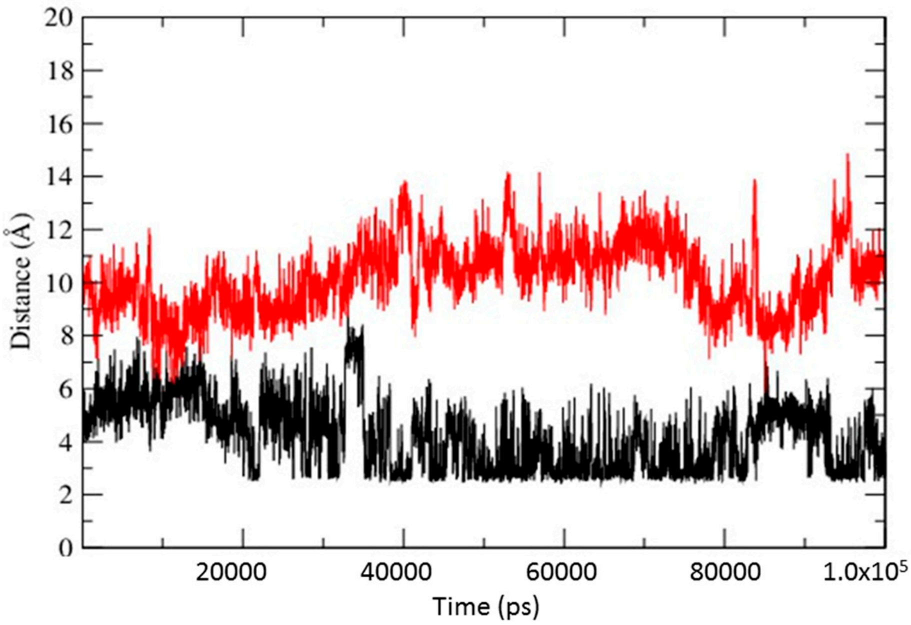

Figure S13. Interactions of the L295S mutant. The side chain of S295 in L295S forms interactions with the backbone of P256 of the linker (black) but not with the side chain of R780 (red) of the THP leading strand.

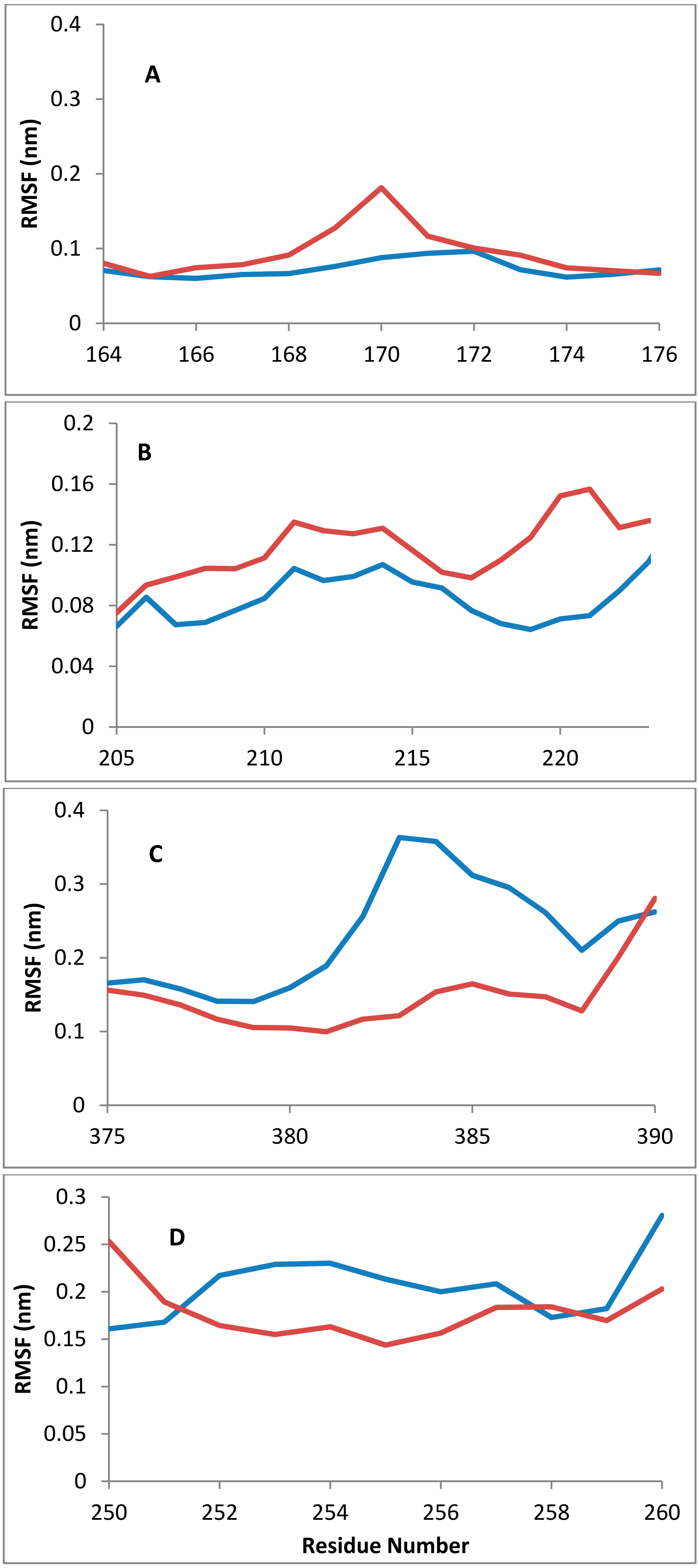

Figure S14. RMSF analysis of the L295S mutant (red) in comparison to WT MMP-1•THP (blue). (**A**) Residues 164–176 belong to β4–β5 of the CAT domain; (**B**) Residues 205–225 belong to loop region between α-helix B and C of the CAT domain; (**C**) Residues 375–390 belong to blade III of the HPX domain; (**D**) Residues 250–260 are part of the linker region.

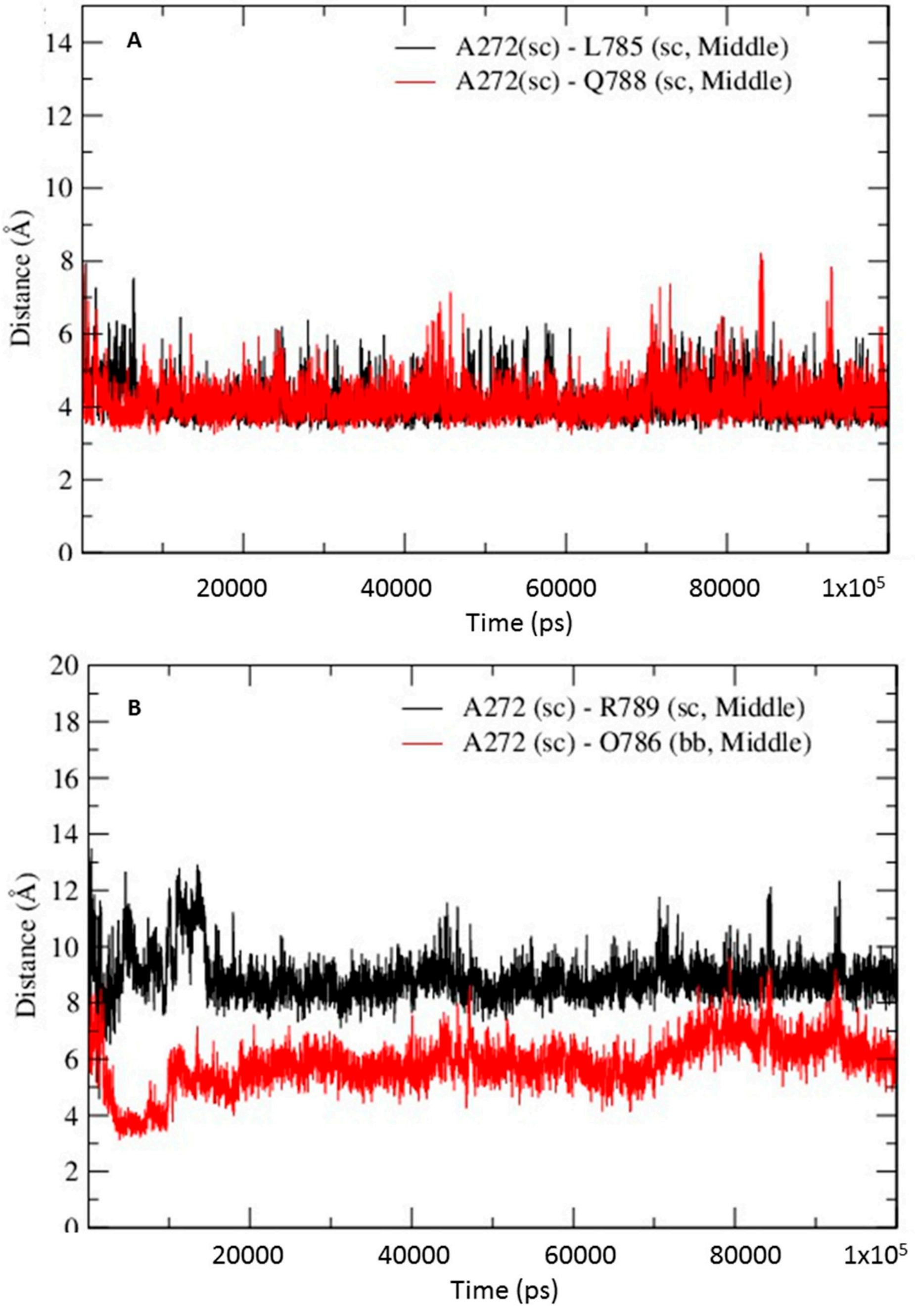

Figure S15. Interactions of the R272A mutant with the THP middle strand. (**A**) Distances to L785 and Q788; (**B**) Distances to R789 and O786.

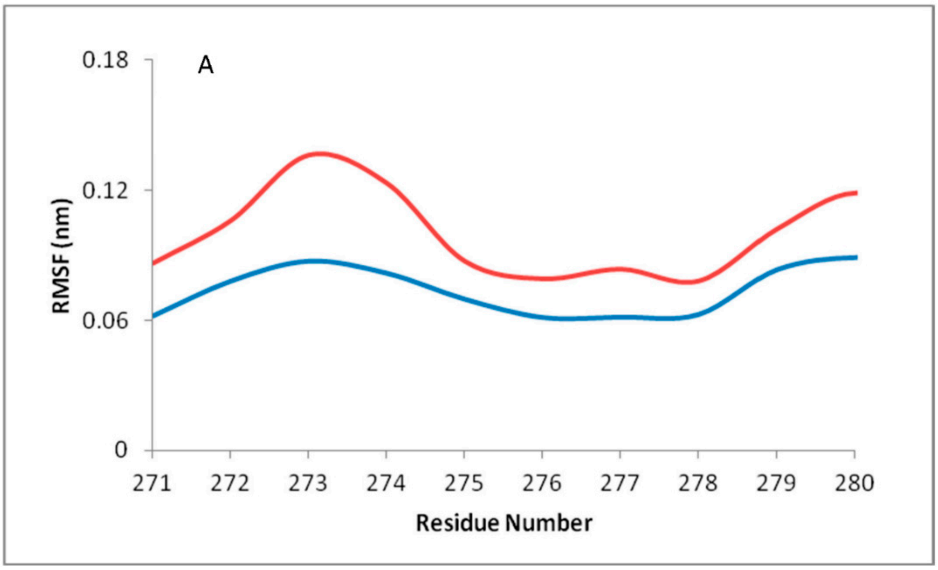

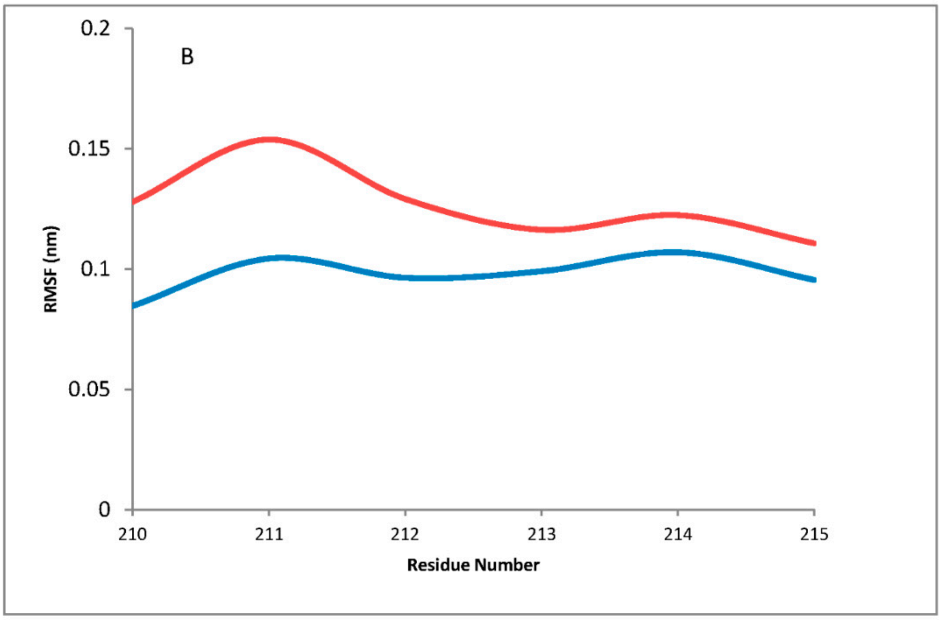

Figure S16. RMSF of R272A mutant (red) in comparison to WT MMP-1•THP (blue). (**A**) Residues 271–280, belonging to the loop between β6–β7 and also part of β7 (HPX domain blade I); (**B**) Residues 210–215, belonging to the loop region connecting α-helix B and C in the CAT domain.

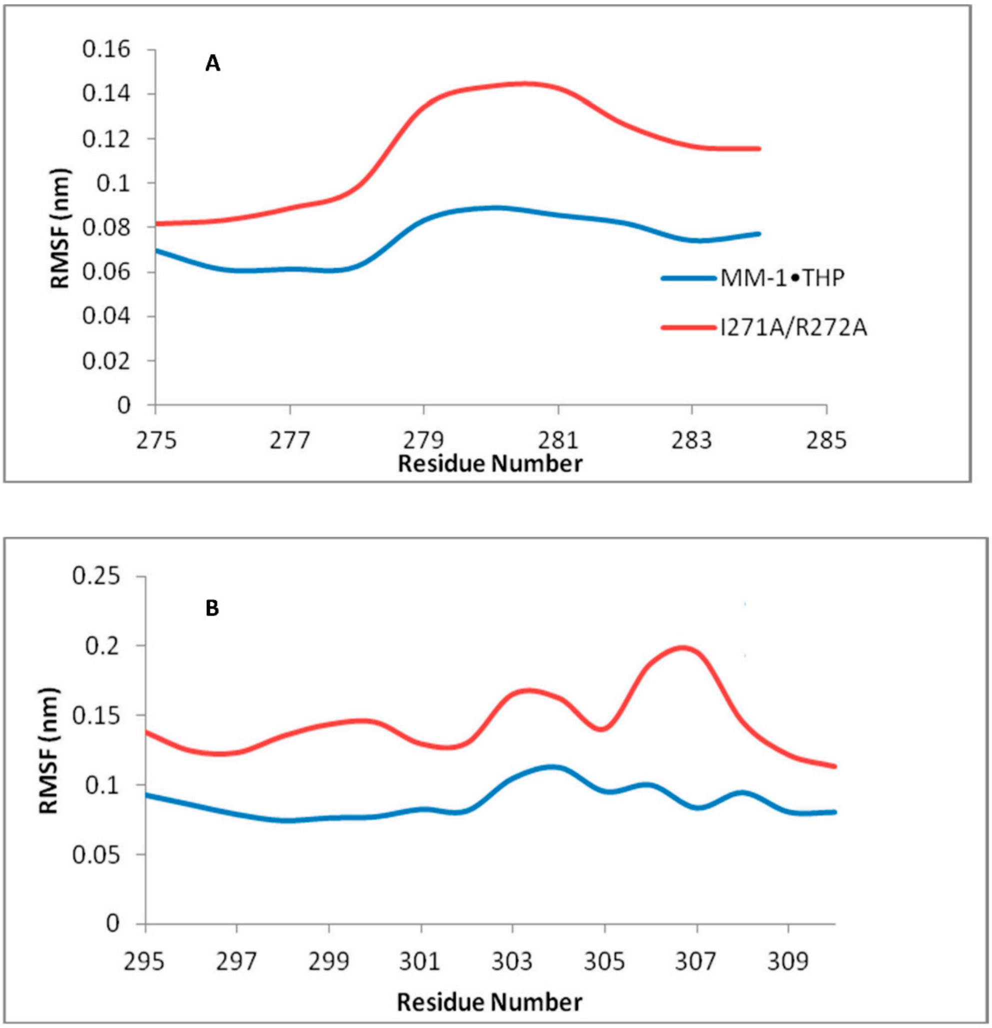

Figure S17. RMSF of I271/R272A (red) in comparison to WT MMP-1•THP (blue). The residues spanning 275–310 belong to blade I in the HPX domain. (**A**) Residues from 275–285; (**B**) Resides from 295–310.
